# Complete Genome Sequence of New *Cronobacter-*Specific Bacteriophage Dev_CS701

**DOI:** 10.1128/mra.00034-23

**Published:** 2023-03-30

**Authors:** Michal Kajsik, Peter Durovka, Maria Kajsikova, Diana Rusnakova, Tomas Szemes, Hana Drahovska

**Affiliations:** a Medirex Group Academy n.o., Nitra, Slovakia; b Comenius University Science Park, Bratislava, Slovakia; Loyola University Chicago

## Abstract

*Cronobacter* is a ubiquitous Gram-negative bacterium linked with serious infections. In this report, we describe the characterization of *Cronobacter* phage Dev_CS701, which was isolated from wastewater. Related to phages belonging to the *Straboviridae* family and *Pseudotevenvirus* genus, such as vB_CsaM_IeB, Dev_CS701 contains 257 predicted protein-coding genes and a tRNA gene.

## ANNOUNCEMENT

Members of *Cronobacter* genus are opportunistic pathogens that can cause infections in neonates, including meningitis, necrotizing enterocolitis, and sepsis (1, 2). *Cronobacter* is also capable of causing infection in the elderly and immunocompromised adults (3). A common source of infection is transmission from food. *Cronobacter* spp. have been isolated from several types of food, but with regard to its pathogenicity towards infants, the biggest problem is caused by its occurrence in infant formula as the contamination of infant formula was associated with infections of newborns (4). Here, the isolation and genome sequencing of bacteriophage Dev_CS701 are reported. Phage Dev_CS701 was amplified from filtered (0.22-μm pore size) wastewater treatment plant samples collected in September 2012 in Bratislava, Slovakia, by growth on Cronobacter sakazakii 701 (5). The host was cultivated aerobically in the same volume of 2-fold-concentrated LB broth and wastewater sample at 37°C. Dev_CS701 was propagated, and host specificity was determined by the double-agar overlay plaque assay (6). Phage was purified by three repeated isolations from single plaques on double agar, amplified in liquid cultures, and further purified by ultracentrifugation in a cesium chloride gradient (7). Phage DNA was purified using a Norgen Biotek phage DNA isolation kit (Norgen Biotek, Thorold, Canada). Next-generation sequencing libraries were generated with a Nextera XT DNA library preparation kit, and phage Dev_CS701 was sequenced on an Illumina NextSeq 2000 platform with paired-end 100-bp reads using P2 reagents and 200-cycle v3 chemistry. FastQC (www.bioinformatics.babraham.ac.uk/projects/fastqc) was used to control the quality of 28,379,925 sequence reads. The Trimmomatic tool v.0.39 was used for trimming reads before assembly using SPAdes v.3.5.0 (8, 9). The assembly resulted in a single contig with 6,059× coverage. To finalize the sequence and ensure that the complete termini will be present, PCR products (forward, 5′-TTTGGGTTTGCTCTGGGTTG-3′; reverse, 5′-CAGGAACGCGCATTCTACTC-3′) amplified off the contig ends were Sanger sequenced.

Protein-coding genes were predicted using GLIMMER v3.0, and a tRNA gene was localized with ARAGORN v.1.2.41 (10, 11). Functional annotations were guided by results from RASTk v.2.0, InterProScan v.5.52-86.0 (12), and BLAST v.2.2.31 (13) analyses. BLAST searches were conducted with the NCBI nonredundant, UniProtKB Swiss-Prot, and UniProtKB TrEMBL databases (14). Unless otherwise stated, all tools were executed using the default parameters. Whole-genome comparisons were performed by the ANI Calculator using the OrthoANIu algorithm (15). The phage life cycle was predicted by the PhageAI tool (16).

Bacteriophage Dev_CS701 was predicted to be virulent with an estimated accuracy of 99.61%. It is capable of infecting and lysing Cronobacter sakazakii strain 701. Dev_CS701 has a 178,510-bp genome with a G+C content of 44.8%. A total of 257 coding sequences and 1 tRNA gene (tRNA-Gly-TCC) were annotated, resulting in a protein-coding density of 94%. Phage Dev_CS701 shares high nucleotide-level similarity with phages from the *Pseudotevenvirus* genus, such as enterophage vB_EkoM5VN (LC589952), cronophage vB_CsaM_GAP161 (NC_019398.1) (17), and cronophage vB_CsaM_IeB (NC_048645) with average nucleotide identities (ANIs) of 96.22%, 96.08%, and 97.70%, respectively.

### Data availability.

The genome sequence of bacteriophage Dev_CS701 was deposited in GenBank under accession no. ON157416 and is available under BioProject accession no. PRJNA922742, SRA accession no. SRR23073236, and BioSample accession no. SAMN32691646.
